# Characterization of C-phycocyanin antioxidant, anti-inflammatory, anti-tumour, and anti-HCoV-229E activities and encapsulation for implementation in an innovative functional yogurt

**DOI:** 10.1016/j.heliyon.2024.e31642

**Published:** 2024-05-31

**Authors:** Tarek Nour Soliman, Asmaa Negm El-Dein, Sahar Abd Al-Diam, Abdou Allayeh, Hanem Awad, Nasser S. Flefil

**Affiliations:** a Dairy Department, Food Industries and Nutrition Research Institute, National Research Centre, 12622, Dokki, Giza, Egypt; b Chemistry of Natural and Microbial Products Department, Pharmaceutical and Drug Industries Research Institute, National Research Centre, 12622, Dokki, Giza, Egypt; c Virology Lab 176, Water Pollution Research Department, Environment and Climate Change Institute, National Research Centre, 12622, Dokki, Giza, Egypt; d Department of Tanning Materials and Leather Technology, National Research Center, Dokki, Giza, Egypt; e National Institute of Oceanography and Fisheries, NIOF, Cairo, Egypt

**Keywords:** C-Phycocyanin, *Spirulina platensis*, Pharmacological properties, Human coronavirus 229E, Nano-phytosome, Functional yogurt

## Abstract

Functional bioactive ingredients isolated from microalgae as sustainable sources have become a new subject of pharmacology and functional foods. Thus, the work aims to produce crude phycocyanin (C-PC), define it, and investigate its pharmacological effects before warping it in a nanophytosome. Subsequently, the physicochemical properties of nanoparticles were evaluated. Both free and nanophytosomes of C-PC were incorporated into cow milk fermented with the probiotic *Lactobacillus rhamnosus* KU985435 to make functional yoghurt and the stability of C-PC of both phytosomes was assessed. The amino acid content of C-PC revealed the presence of eight of nine essential amino acids and eight of eleven non-essential amino acids. C-PC has a medium molecular weight (82.992 kDa). Some pharmacological effects like reducing inflammation (98.76 % ± 0.065), fighting free radicals (99.12 % ± 0.027), and being able to inhibit the human coronavirus 229 E with a selective index of 27.9 were observed. The maximum viral inhibitory activity was detected during the adsorption stage. Anti-human liver and colon carcinomas that exceeded Doxorubicin with very low cytotoxicity against normal cell lines were detected. C-PC is an unstable protein that could be degraded in the yoghurt during storage. Therefore, phytosome encapsulation can effectively stabilize C-PC (particle size 44.50 ± 12 nm and zeta-potential −32.4 ± 5 mV) and protect it from the acidic environment of the yoghurt. The produced yoghurt showed the desired physicochemical and functional properties and overall acceptance. The results prove that C-PC from spirulina algae is a renewable source of dyes. The encapsulation process using phytosomes gave it high stability against environmental influences, and therefore, it can be applied in the food and pharmaceutical industries in the future.

## 1 Introduction

The search for extracted and purified microalgal substances with nutraceutical and health benefits has attracted the interest of the food and biotechnology industries and a lot of researchers [[1], [2], [3]]. C-phycocyanin (C-PC) is a photosynthetic water-soluble pigment protein found in cyanobacteria, cryptophytes, rhodophytes, and glaucophytes [4]. Like other pigments, such as phycoerythrin, C-PC is categorized as a phycobiliprotein (PBP). C-PC has been extensively studied and has a variety of uses in the food, cosmetics, pharmaceutical, and biotechnology industries. C-PC has been primarily employed as a natural dye and is a good alternative to hazardous and carcinogenic chemical colorings in the food sector due to its blueness and functional characteristics [5]. C-PC also has a wide range of pharmacological benefits, including antioxidant, anti-inflammatory, anticancer, and health-promoting potentials, as well as a decrease in photo-induced cytotoxicity and immune system stimulation [[6], [7], [8], [9], [10]]. C-phycocyanin also protects the liver by preventing hepatic lipid peroxidation, functioning as an antioxidant [11]. C-phycocyanin also scavenges free radicals from damaged nerve cells, preventing DNA oxidative damage and neuronal cell death caused by free radicals [12,13]. C-PC is a photosensitive and thermos-sensitive protein susceptible to denaturation at temperatures exceeding 45 °C, which leads to color changes.

Nanoencapsulation prolongs the half-life of sensitive compounds and improves their absorption capabilities inside the body [14]. Nanoencapsulation allows for the controlled release of these substances, preserves the taste and color properties of bioactive compounds, and enhances the solubility of hydrophobic molecules in aqueous solutions. Nanoliposomes enhance the efficacy of bioactive pharmaceuticals by mitigating unfavorable interactions between bioactive molecules and enhancing their solubility, bioavailability, and stability both *in vitro* and *in vivo* [15]. Phytosomes are liposomal structures formed by combining natural phospholipids with phytochemicals in a suitable solvent. Phospholipids, specifically phosphatidylcholine, mix with polyphenolic chemicals to create nano-pyrosomes due to their dual roles. Phosphatidylcholine's hydrophobic chains combine in an aqueous environment to form a hydrophobic bilayer. The external surface is composed of the hydrophilic choline structure, facing towards water. Spherical nanostructures originate in the water-based environment inside and outside the lipid bilayer interface [16].

Functional fermented products such as yoghurt have recently got much attention, mainly because living standards have grown and health issues have become more prominent. Because of the flavor enhancement and health benefits acquired after consuming these probiotics, fermentation of food products with lactic acid bacteria (LAB) is the most prevalent fermentation. Foods, drinks, and by-products from food processing can benefit from LAB by increasing texture and nutraceutical profile [[17], [18], [19]]. Lactic acid fermentation improves food safety, shelf-life stability, sensory properties, and nutritional qualities [[20], [21], [22], [23]]. Yoghurt's health benefits may be attributed to the culture consortia that participate in the fermentation process, biochemical changes fermentation produces in milk, or both. Fermentation-related microbial cultures are diverse and provide the product with unique functional characteristics. Yoghurt that combines fresh tastes and colors with possible health advantages boosts sales and improves customer satisfaction. Incorporating phycocyanin, which has antibacterial qualities and influences its physicochemical characteristics, may suppress yoghurt cultures [24]. Therefore, this work investigates the production of locally crude phycocyanin by a novel technique and verifies its antioxidant, anti-inflammatory, anti-tumor, and *anti*-HCoV-229E activities. Moreover, the food sector cannot use C-PC due to its susceptibility to denaturation and/or proteolysis, so it is encapsulated in a complex nanophytosome. The C-PC-loaded phytosome was applied as a natural colorant in a highly biologically active, innovative, functional yoghurt. The stability of C-PC during processing and its ability to maintain the shelf-life of yoghurt were assessed.

## 2 Materials and methods

### 2.1 Materials

All materials used for bacterial or algal propagation and the listed chemicals were purchased from Sigma Aldrich Co. (St. Louis, MO, USA) and stored at room temperature until otherwise stated. Materials used for antioxidant (DPPH, ABTS) and antitumor (MTT) activities were stored at −20 °C. Materials used for yoghurt preparation were of food grade and stored in a refrigerator.

#### 2.1.1 Algal strain

The blue-green alga, *S. platensis,* used in the present study was obtained from the Freshwater Hydrobiology Lab, National Institute of Oceanography and Fisheries (NIOF), Cairo, Egypt.

### 2.2 Methods

#### 2.2.1 Medium and growth conditions

Zarrouk medium was used for culturing *S. platensis* for 21 days [25,26]. *S. platensis* was grown in a batch culture at 32 °C ± 2 °C, supported by a continuous air pump for aeration, with adjusted pH 9, and light intensity ∼2000 lux from a fluorescent lamp for 24-h lighting. The cells were allowed to grow to a density of about 1.0 mg ml^−1^ and were harvested by centrifugation.

#### 2.2.2 Extraction of C-phycocyanin

*S. platensis* biomass slurry was firstly washed with double distilled water to remove adhered salts then grounded manually (in the dark) in 10 mL of 0.1 M phosphate buffer solution (pH: 6.8) [27], and 1 mL of Tris HCl in the presence of acid-washed neutral sand, and then filtered. The mixture was further subjected to freezing (−20 °C for 30–100 min) and thawing for 3–10 freeze-thaw cycles in total [28], stirred at 150 rpm at 4 °C for 30 min, sonicated ten times at 40 °C, 50/60 HZ, 195 W with 10-s sonication and 10-s intervals (Ultrasonics-HD, J.P. Selecta, S.A., Spain), centrifuged at 4 °C for 8 min at 3823×*g* (6000 rpm) (Sigma, 2–16P, UK), and the blue-colored supernatant was taken for further investigations.

C-phycocyanin calculations were determined using spectrophotometry-based methods on the absorbance ratio (Cary 3500 UV–Vis, Agilent T, England).

The content of C-phycocyanin was calculated according to Setyoningrum et al. [29] C-phycocyanin has a single visible absorbance maximum between 615 and 620 nm.

The calculations were done according to equation (1):

(1) $$\%\;C-PC=\left(\frac{{\mathrm{OD}}_{620}\times V\times 100}{3.39\times W2\times Dw}\right)$$

where C-PC is the crude C-phycocyanin (%), A_620_ represents the absorbance of C-phycocyanin at 620 nm, 3.39 is the extinction coefficient of C-PC at 620 nm, V is the total volume, 100 represents 100 %, W2 is the weight of wet biomass, and DW represents the percentage of dry weight.

According to equation (2), the determination of C-phycocyanin purity is based on the absorbance ratio A620/A280 nm, which corresponds to C-phycocyanin and total protein, respectively [30,31]**.**

(2) $$Purity\;ratio\;of\;C-PC=\frac{A_{620}}{A_{280}}$$

#### 2.2.3 Fourier-transform infrared spectroscopy (FT-IR) analysis of C-phycocyanin

An infra-red spectrum of C-PC was obtained using a Fourier transform infrared spectrophotometer (JASCO FT/IR 4600 FT-IR, Germany), as it is widely used technique to study protein secondary structure both in solution and in the solid state [32]**.** The sample was ground with spectroscopic grade KBr powder and then pressed into 1 mm pellets for Fourier-transformed infrared (FT-IR) measurement in the frequency range of 4000–400 cm^−1^ (Mid-infrared region).

#### 2.2.4 Molecular weight determination of C-phycocyanin by GPC analysis

The C-PC was applied to gel permeation chromatography (GPC, Water Alliance 2695, Water Corporation, USA) to determine its molecular weight [33]**.** The sample was dissolved in deionized water, filtrated through a 0.45 mm Millipore membrane filter (Axiva, China), and applied to the GPC water column (PL aquagel-OH 7.5 mm, 50 μm pore type, 8 μm particle size).

#### 2.2.5 Amino acid analysis of C-phycocyanin

To extract amino acids from C-PC, 100 mg of the sample was mixed with 5 mL H2O and 5 mL of 6 M HCl, heated at 120 °C for 24 h, and filtered. Finally, 1 ml of the filtrate was injected into HPLC. The amino acid content of C-PC was analyzed via Agilent 1260 series HPLC analysis according to Campanella et al. and Jajić et al. [34,35]**.**

#### 2.2.6 DPPH and ABTS radical scavenging activity of C-phycocyanin

The DPPH (1–diphenyl–2–picrylhydrazyl) scavenging activity of C-PC was measured by the method of **Lee et al.** [10]. 500 μl of ethanolic DPPH solution (0.4 mmol) was mixed vigorously with 500 μl of the sample (equivalent to 0.005 mg) separately or water (as a control) and incubated at 37 °C in the dark for 1 h. The absorbance of the mixture was measured spectrophotometrically at 517 nm. The scavenging activity was calculated according to equation (3);

(3) $$\%\;Scavenging\;activity=1-\left(\frac{As-Ab}{Ac}\right)100$$

where Ab, Ac and As are the absorbance of the blank (ethanol and sample), the control (DPPH and deionized water), and the sample (DPPH and sample), respectively. This experiment was conducted in duplicate; all values are expressed as means ± standard deviation and compared to the control (ascorbic acid at the concentration of 0.1 %).

The ability of the sample to scavenge 2,2′-azino-bis(3-ethylbenzothiazoline-6-sulfonic) acid (ABTS) radical cation was performed as described by **Re et al.** [36]**.** The radical cation was prepared by combining a 7 mM ABTS stock solution with 2.45 mM potassium persulfate (1:1 (v/v)) and leaving the mixture for 4–16 h until the reaction was complete and the absorbance was stable. For measurements, the ABTS solution was diluted with ethanol to achieve an absorbance of 0.700 at 734 nm. After mixing 0.9 mL of ABTS solution and 0.1 mL of sample for 45 s, measurements at 734 nm were taken after 30 min. The antioxidant activity of the samples was calculated by determining the decrease in absorbance by using equation (4):

(4) $$\mathrm{Scavengin}g\;activity\;\%=\left(\frac{Ac-At}{Ac}\right)*100$$

where At and Ac are the corresponding absorbances of the examined samples and ABTS.

#### 2.2.7 Anti-inflammatory activity using the human red blood cell (HRBC) membrane stabilization method

HRBC method was used to determine the anti-inflammatory activity of C-PC as described in the literature [37]. The blood sample was collected from a healthy volunteer who was not taking any non-steroidal drugs two weeks before the experiment. The blood was mixed with an equal volume of sterilized Alsever solution (2 % dextrose, 0.8 % sodium citrate, 0.05 % citric acid, and 0.42 % NaCl in water). After centrifuging the reaction mixture at 3000 rpm, the packed cells were isolated, and 10 % v/v suspension was made with iso-saline. The HRBC suspension was used for the estimation of the anti-inflammatory properties. Standard drugs, Aspirin and Diclofenac were used as the reference.

100 μl of C-PC solution (equivalent to 0.001 gm) and control (water) were separately mixed with 1 ml of phosphate buffer, 2 ml of hyposaline, and 0.5 ml of HRBC solution. All the assay mixtures were kept in incubation for 30 min at 37 °C and then centrifuged at 3000 rpm for 10 min. The hemoglobin content in the supernatant was estimated by a spectrophotometer at 560 nm. The percentage of hemolysis was estimated by assuming the hemolysis produced in control as 100 %.

The percentage of protection was calculated using equation (5):

(5) $$\%\;Protection=100-\left(\frac{As}{Ac}\right)*100$$

where As and Ac are the corresponding absorbance of the examined samples and control.

#### 2.2.8 Antiviral activity of C-phycocyanin

##### 2.2.8.1 Viruses and cells

A viral strain and a cell line were obtained from the American Type Culture Collection (ATCC, USA) and generously donated by Nawah-Scientific Co., Egypt. For the propagation of human coronavirus 229E (HCoV-229E), a clone of Vero (Vero-E6) cells was used as the host cell line. The cells were cultured in DMEM-high glucose medium containing 10 % fetal bovine serum, 0.1 % antibiotic solution (Gibco BRL, NY, USA), and trypsin-EDTA (Sigma-Aldrich, USA).

#### 2.2.9 Cytotoxicity assessment

Vero–E6 cells were inoculated at 2 × 10^4^ cells per well in 96-well plates to test the C-phycocyanin's cytotoxicity. On Vero–E6 cell monolayers grown in 5 % CO_2_, 37 °C, the cytotoxicity of the C-phycocyanin was measured. After removing the growing media, ten-fold dilutions of the C-phycocyanin in 0.01 mL of medium were added and cultured for 72 h at 37 °C in a 5 % CO_2_ environment. Each concentration was performed in three replicates. Cells from wells that were not subjected to compound treatment served as normal controls. The supernatant was aspirated, and the cell monolayers were washed with 0.03 mL of sodium chloride solution to eliminate unviable cells before the staining procedure with crystal violet based on the principles stated by **Nain et al.** [38]. The cells were then fixed and stained in a single step using 100 μl of 0.03 % crystal violet (w/v) in 50 % methanol for 10 min, followed by 300 μl of water washings, then the stained cells were treated for 20 min with 100 μl lysis buffer (0.8979 g sodium citrate and 1.25 ml 1 N HCl in 98.05 ml 47.5 % ethanol). The optical density of wells was measured using a spectrophotometer at 540/620 nm (BMG LabTech, Ortenberg, Germany). Cell viability was calculated as a percentage of the mean value of optical density obtained from cell controls, which was set at 100 %. Using the GraphPad PRISM software, the 50 % cytotoxic concentrations (CC_50_) were determined from the mean dose-response curves.

##### 2.2.9.1 Cytopathic effect inhibitory assay

The cytopathic effect inhibitory (CPE) test uses human coronavirus 229E to lyse Vero–E6 cells and identify substances effective at any step of the viral multiplication life. The antiviral assessment of the assay is frequently performed by rating the CPE microscopically. Crystal violet was used in this study to produce more objective findings. The CPE inhibitory experiment, like the cytotoxic assay, was performed in 96-well plates with Vero–E6 cells. Following aspiration of the cell growth medium, 50 μl of the C-phycocyanin was diluted in the medium (the same concentrations used in the cytotoxicity assay) and a constant multiplicity of infection (MOI) of the HCoV-229E in 50 μl of the appropriate medium was added to the wells. On each plate, non-infected and infected cells without C-phycocyanin served as cell and viral controls, respectively. Plates were incubated at 37 °C in 5 % CO_2_. Inverted microscopy was used to track the progression of the cytopathic effect. In terms of virus-induced CPE inhibition, the untreated infected control had the greatest cytopathic impact. At this stage, the cell was fixed and stained with a 0.03 % crystal violet solution in 2 % ethanol and 3 % formalin in water, using the protocol for the cytotoxicity experiments. **Pauwels et al.** [39] used equation (6) to compute the percentage of antiviral activity of the tested compound:

(6) $$Antiviral\;activity\;\left(\%\right)=\frac{\left(mean\;optical\;density\;of\;control\;cells-mean\;optical\;density\;of\;treated\;cells\right)}{mean\;optical\;density\;of\;control\;cells}*100$$

based on these findings, the 50 % CPE inhibitory dosage (ID_50_) was estimated.

#### 2.2.10 Time of addition assays

The cells were treated with C-phycocyanin before, during, and after viral infection. To assess the manner of antiviral activity, the maximal non-cytotoxic concentration was always employed. Before viral injection, cells were pretreated with C-phycocyanin by adding the C-phycocyanin to the medium and incubating for 1 h at 37 °C. For C-phycocyanin pretreatment of HCoV-229E, 2.5 × 10^3^ of HCoV-229E were cultured in media containing the C-phycocyanin for 1 h at room temperature before infection of Vero-E6 cells. The effect of C-phycocyanin on HCoV-229E replication was also investigated by adding C-phycocyanin after cell infection. Each experiment was performed three times. CPE-inhibition experiments were performed as described previously, and the percentage of inhibition of C-phycocyanin-treated cells and viruses was computed and compared to untreated controls. As controls, wells with media containing 1 % ethanol with no C-phycocyanin were put on each plate [40,41].

#### 2.2.11 Antitumor activity of C-phycocyanin

##### 2.2.11.1 Cell culture conditions

Human colorectal carcinoma (HCT-116), human liver carcinoma (HepG-2), MCF-7 (human breast adenocarcinoma), and the normal human skin fibroblast (BJ-1) cell lines were purchased from the American Type Culture Collection (Rockville, MD, USA) and maintained in Dulbecco's Modified Eagle's Medium (DMEM) supplemented with 10 % heat-inactivated fetal bovine serum (FBS), 100 U ml^−1^ penicillin, and 100 U ml^−1^ streptomycin. The cells were grown at 37 °C in a humidified atmosphere of 5 % CO_2_.

##### 2.2.11.2 MTT anti-proliferative assay

The anti-proliferative activity on the HepG-2 (human liver carcinoma), HCT-116 (Human colorectal carcinoma), MCF-7 (human breast adenocarcinoma) and BJ-1 (normal human skin fibroblast) was estimated by the 3-[4, 5-dimethyl-2-thiazolyl)-2, 5-diphenyl-2H-tetrazolium bromide (MTT) assay. This test is based on MTT cleavage by mitochondrial dehydrogenases from viable cells [42]. Cells were placed in a 96-well sterile microplate (5 × 10^4^ cells well^−1^) and incubated at 37 °C in serum-free media containing dimethyl sulfoxide (DMSO) and either a series of various concentrations of each sample or Doxorubicin (positive control) for 48 h before the MTT assay. After incubation, the media were removed and 40 μL MTT (2.5 mg ml^−1^) was added to each well. Incubation was resumed for an additional 4 h. The purple formazan dye crystals were solubilized with 200 μL DMSO. Absorbance was measured at 590 nm in a Spectra Max Paradigm Multi-Mode microplate reader (Molecular Devices, LLC, San Jose, CA, USA). Relative cell viability was expressed as the mean percentage of viable cells compared to the untreated control cells. All experiments were conducted in technical triplicate and three biological replicates. All values were reported as mean ± SD. IC_50_ was determined by SPSS Incprobit analysis (IBM Corp., Armonk, NY, USA).

### 2.3 Preparation of C-PC nanophytosomes

The thin layer hydration approach was used to encapsulate C-PC into nanophytosomes with different ratios of C-PC and soy lecithin (1:1, 1:2, and 1:3) [43]. C-PC (500 mg) and lecithin (500, 1000, or 1500 mg) were weighed and dissolved in ethanol before being inserted into a vacuum rotary evaporator (Heidolph, Laborota 4002 control, Germany) in a round bottom flask for 60 min at 60 °C to remove all of the organic solvents and form a thin, dry film at the bottom of the flask. In a rotary at 60 °C, the corresponding thin layer was hydrated with 100 mL of distilled water. The size of the initial phytosomes was reduced using a combination of homogenization (Sonic Vibra cell, Newton, CT, USA) at 20,000 rpm for 20 min, followed by ultrasonication for 15 min at 160 W power, 40 kHz frequency, and 50 percent pulse (Newtown, CT, USA). Temperature rises during ultrasonication were reduced by putting the sample container in a large beaker filled with ice.

#### 2.3.1 Characterization of C-PC nanophytosomes

The dynamic light scattering (DLS) technique was used to examine the surface charge (zeta potential), mean particle size diameter, and polydispersity index (PDI) of C-PC nanophytosomes (Zetasizer Nano Z.S., Malvern Instruments, UK). Transmission electron microscopy (TEM) was utilized to analyze and observe the morphology of nanophytosomes. A JEOL JEM-1400 plus TEM with an accelerating voltage of 100 kV and a magnification of 200,000× was utilized [43].

#### 2.3.2 Loading properties

The centrifugation technique was used to determine the encapsulation efficiency (EE) and loading capacity (LC) of C-PC in nanophytosomes [44]. 2 mL phytosomal formulation plus 2 mL DW was homogenized. The sample was then centrifuged for 30 min at 4 °C at 15,000×*g* (Eppendorf centrifuge 5804 R, Hamburg, Germany). The quantity of C-PC was evaluated by spectrophotometer using the calibration curve (in the concentration range of 1–15 μg/mL and R^2^ = 0.9706 (n = 6)) and measuring the absorbance at max = 620 nm (Cary 3500 UV–Vis, Agilent T, England). The following equations (7), (8)) were used to calculate EE and LC:

(7) $$EE\;\left(\%\right)=\frac{Amount\;of\;encapsulated\;phycocyanin-\;Amount\;of\;free\;phycocyanin}{Amount\;of\;encapsulated\;phycocyanin}*100$$

(8) $$LC\;\left(\%\right)=\frac{Amount\;of\;encapsulated\;phycocyanin-\;Amount\;of\;free\;phycocyanin}{Amount\;of\;carrier\;\left(lecithin\right)}*100$$

### 2.4 Production of a functional probiotic-fermented stirred yoghurt supplemented with free and nanophytosomes of C-phycocyanin

#### 2.4.1 Probiotic strain used in yoghurt preparation

*Lactobacillus rhamnosus*, a potent probiotic previously identified and deposited in Gene Bank under accession number KU985435**by Negm El-Dein et al.** [45], was used in yoghurt preparation. The bacterial strain was routinely cultivated at 37 °C on an MRS medium and stored in 50 % (v/v) glycerol at −80 °C. Before inoculation into pasteurized skim milk, cells of *L. rhamnosus* were washed twice with sterilized ultrapure water and adjusted to 1 at OD_600_ to ensure the number of surviving bacteria in the preparations (10^6^–10^8^ CFU/ml), inoculated (10 % v/v) into the milk and incubated for 6 h at 45 °C.

#### 2.4.2 Production of a functional stirred yoghurt

Cow milk was heated to 85-90 °C for 5 min and cooled to 42 °C. It was inoculated with 3 % of starter cultures and then incubated at 45°C for 3 h until a firm curd was obtained. Then, the curd was refrigerated at 4 °C overnight before being divided into five portions. The first portion was kept as a control (C), while two portions were fortified with 150 and 300 mg free C-PC, respectively and stirred using the mixer to prepare T1 and T2. The last two portions were fortified with 150 and 300 mg C-PC encapsulated in phytosome (1:2) which exerted good PDI, size and surface charge, respectively to give T3 and T4. The stirred yoghurts were all enhanced with 0.75 % blueberry flavor. After that, they were kept in a refrigerator at 5 ± 1 °C, and their chemical, rheological, and sensory qualities were examined.

##### 2.4.2.1 Physicochemical properties of the stirred yoghurt

A pH meter and ATC probe combination were used to test the pH of the stirred yoghurt samples (model IQ 240, IQ Scientific Instruments Inc., San Diego, Calif., USA). In addition, acidity was tested according to the **AOAC** [46]. The apparent viscosity of the stirred yoghurt was measured according to **Soliman and Shehata** [47] using a Bohlin coaxial cylinder viscometer (Bohlin Instrument Inc., Sweden) attached to a workstation loaded with the software of the V88 viscometry program. The viscometer probe, system C30, was placed in the yoghurt sample cup, and viscosity measurements were carried out at 20 °C ± 2 °C in the up mode at shear rates ranging from 37 to 1238 1/s.

##### 2.4.2.2 Sensory evaluation of the stirred yoghurt

The stirred yoghurt samples were evaluated for flavor (45 points), body and texture (40 points), and color and appearance (15 points) by 15 panelists from the staff members of the Dairy Department, National Research Centre, Dokki, Giza, Egypt. The scorecard was designed as described by **Soliman and Shehata** [47].

##### 2.4.2.3 Determination of shelf-life stability of the stirred yogurt

###### 2.4.2.3.1 Extraction of total polyphenols in yoghurt samples

The extraction of total polyphenols from yoghurt samples was conducted as described by **Moldovan et al.** [48]. Briefly, 10 ml of yoghurt sample was stirred with an equal volume of ethanol: water mixture (60:40) at room temperature for 30 min. The resulting mixture was centrifuged in a cooling centrifuge (4 °C, 5000 rpm for 15 min). The collected supernatant was then stored at 2 °C and further used to evaluate the samples’ total phenolic content and antioxidant activity.

###### 2.4.2.3.2 Determination of total polyphenols (TP) and antioxidant capacity

The total polyphenols (TP) content was determined as described by **Fiori et al.** [49] as a colorimetric method using the Folin-Ciocalteu reagent. The 0.5 ml of sample was mixed with 0.5 ml of 10-fold-diluted Folin-Ciocalteu reagent. After 3 min, 4 ml of 7.5 % sodium carbonate was added. The mixture was allowed to stand for 30 min in the dark at room temperature before the absorbance was measured at 725 nm using a spectrophotometer (model 2010, Cecil Instr. Ltd., Cambridge, UK). A calibration curve was made with standard solutions of gallic acid in the range of 0.01-1.00 mg The final results were expressed as milligrams gallic acid of equivalent per gram of dry weight (DW). The total antioxidant capacity of stirred yoghurt was determined as described previously.

##### 2.4.2.4 Viability of probiotic strains in yoghurt samples

The total count of colony-forming units (CFU) of LAB used in preparing yoghurt was monitored periodically for 14 days as illustrated by **Negm El-Dein et al**. [50]. The yoghurt samples were preserved in the refrigerator and lactic acid bacteria are normally psychotropic and can grow at refrigerator temperature. Yoghurt contents were thoroughly mixed with a sterile spatula, serial dilutions were prepared and 20 μl of the suitable dilution was cultured in 20 ml MRS agar. The bacterial count was determined after 24 h of incubation by plate count agar.

### 2.5 Statistical analysis

All data was expressed as mean ± SD. Statistical analyses were carried out by one way analysis of variance (ANOVA), coupled with Minitab software (version 20, India). Statistical difference between groups was considered at (p ≤ 0.05). GraphPad PRISM (Version 8.0.1, San Diego, CA, USA) was used to perform the calculations and illustration of figures.

## 3 Results and discussion

### 3.1 Concentration and purity of C-phycocyanin

*Spirulina* biomass was harvested after 21 days of growth. 15 g wet biomass was dried and yielded about 4 g dry weight. The C-phycocyanin concentration was 19.25 %, C-phycocyanin purity ratio was found to be of reagent grade (0.15). **Gorgich et al.** [51] separated and purified C-phycocyanin from *Spirulina* and obtained a purity ratio of 0.34. When A_620_/A_280_ ≤ 0.7, C-phycocyanin is considered to be of food grade, when 0.7 ≤ A_620_/A_280_ ≤ 3.9, C-phycocyanin is considered to be of reagent grade and when A_620_/A_280_ ≥ 4.0, C-phycocyanin is considered to be of food grade [52].

### 3.2 Fourier-transform infrared spectroscopy (FTIR) analysis of C-phycocyanin

**Fig. 1:**
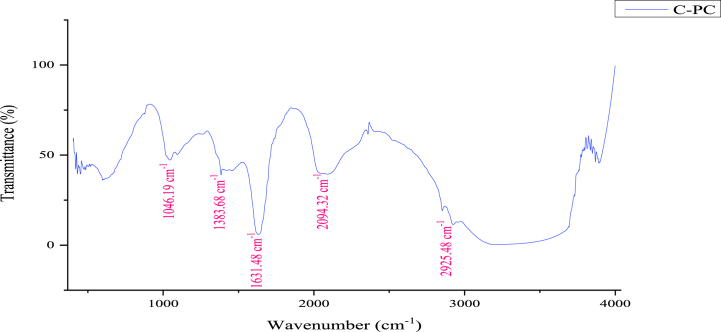
The FT-IR spectrum of *S. platensis* C-PC.

FTIR spectroscopy is a recommended and prevailing analytical method for investigating the structural characterization of proteins in near 1000–1700 cm^−1^. Proteins show high absorbencies in this region, which is within the mid-infrared region and is different for each protein, where the position and the intensity of the bands are specific for every protein allowing its possible identification [53]. The absorbance spectrum of C-PC (Fig. 1) shows bands near the amide I (1844.6 cm^−1^), amide II (1521.6 cm^−1^), and amide III (1285.3 cm^−1^) regions. The modes most widely used in protein structural studies are amide I, amide II, and amide III, with possible shifts to lower frequency in some proteins. The amide I band arises principally from the C=O stretching vibration of the peptide group. The amide II band is primarily N–H bending, contributing from C–N stretching vibrations. The amide III absorption is normally very weak in the infrared, arising primarily from N–H bending and C–N stretching vibrations [53]. This could verify the protein nature of the extracted C-PC.

### 3.3 Molecular weight determination of C-PC by GPC analysis

**Fig. 2:**
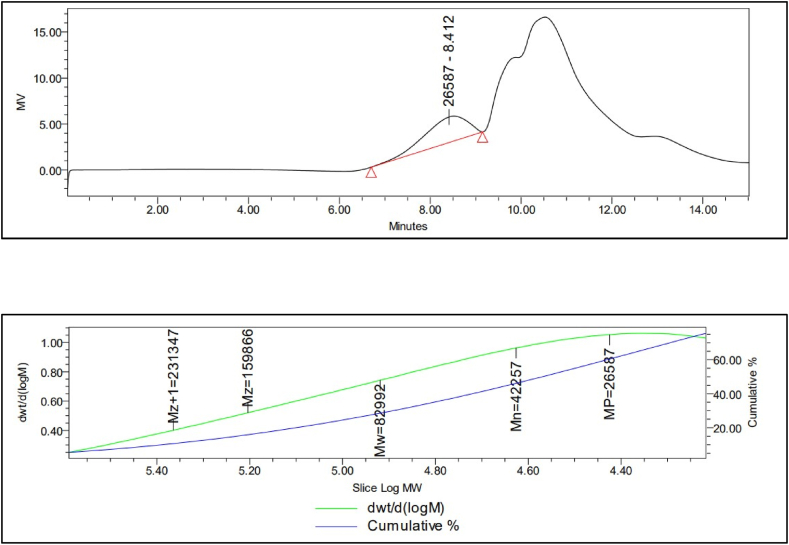
Molecular weight of C-phycocyanin.

One of the most important physicochemical properties of organic compounds is determination of their molecular weight. Gel permeation chromatography (GPC) is one of the most relative and accurate techniques for molecular weight determination. Using the calibration curve of the elution retention time, the molecular weight of C-PC was approximated to be 82992 Da. Fig. 2 presents the chromatogram output of GPC of the tested C-phycocyanin. There is one peak that can be translated to one molecular weight. The polydispersity index (PDI) of the C-phycocyanin peak is 1.964 (Table 1).

**Table 1:** Gel permeation chromatography results of C-PC.

Retention time	Mn	Mw	MP	Mz	Poly-dispersity index
8.412	42257	82992	26587	159866	1.964

Mn: is the number averaged molecular weight; Mw: is the weight averaged molecular weight; Mz: is Z Average or Size Average Molecular Weight.

These results are in agreement with **Hamouda and El-Naggar** [54] who stated that C-phycocyanin possesses a molecular weight ranging from 70 to 110 kDa. It exhibits an average fluorescence emission at approximately 650 nm and a single absorption peak in the visible spectrum, specifically between 615 and 620 nm. C-phycocyanin consists of two subunits, α and β, which are found in equal quantities. However, the exact quantity of α and β pairings that result in the molecular form can vary among different species.

On the other hand, **Patel et al.** [55] reported that the pure C-PC from *Spirulina*, *Phormidium*, and *Lyngbya* spp. had estimated molecular weights of 112, 131, and 81 kDa, respectively.

### 3.4 Amino acid analysis of C-phycocyanin

HPLC analysis of the amino acid content of C-PC (Table 2) revealed the presence of eight out of nine essential amino acids (15.54 μg/g) and eight out of eleven non-essential amino acids (26.46 μg/g). The major essential amino acid is leucine (3.96 μg/g). However, glycine is a significant non-essential amino acid in dried powder (12.26 μg/g). These amino acids are required for making up proteins needed for the repair, growth, and maintenance of cells. Tryptophan and cysteine were not detected in C-phycocyanin. **Ridine et al.** [56] stated that tryptophan and cysteine were absent in the amino acid content of C-phycocyanin. This wide range of amino acids indicates the great biological importance of C-PC proteins. An optimal result might be obtained by adding C-PC enriched bylysine and/or histidine, as well as sulfur-containing amino acids into food matrix.

**Table 2:** Amino acid content of C-PC.

Essential amino acids	Concentration (μg/g)	Non-essential amino acids	Concentration (μg/g)
Histidine	1.53	Asparagine	0.37
Threonine	0.64	Glutamine	2.19
Valine	2.94	Serine	1.16
Methionine	1.21	Glycine	12.26
Tryptophan	0.00	Arginine	3.15
Phenylalanine	3.09	Alanine	2.97
Iso-Leucine	1.64	Tyrosine	0.98
Leucine	3.96	Cysteine	0.00
Lysine	0.53	Proline	3.38

### 3.5 Antioxidant and anti-inflammatory activity of C-phycocyanin

In this study, *in vitro* antioxidant and anti-inflammatory assays were applied to evaluate the C-PC activity. C-PC was able to scavenge DPPH free radicals by antioxidant activity up to 99.12 ± 0.027 % compared to the reference standard (Table 3). HRBC membrane stabilization method was used to evaluate the anti-inflammatory activity of C-PC, which revealed activity up to 98.76 ± 0.065 compared to the reference standards (Table 3). The HRBC membrane stabilization method is usually used to investigate the anti-inflammatory properties of HRBCs because RBCs resemble lysosomes in terms of membrane similarity, which was first noted by **Chou** [57], because of the intricate association of lysosome rupture with the consequences of inflammation [58]. In both methods, activity was observed at relatively low concentrations of C-phycocyanin. Potent antioxidant and anti-inflammatory activities were reported for C-PC [7,59]**.**

**Table 3:** Antioxidant and anti-inflammatory activities of C-PC.

Sample	Antioxidant (%)	Anti-inflammatory (%)
C-phycocyanin	99.12^a^ ±0.027	98.76^a^ ±0.065
Ascorbic acid	100^a^ ±0.00	–
Aspirin	–	97.90^ab^ ± 0.173
Diclofenac	–	99.51^a^ ±0.043

### 3.6 Antiviral activity of C-phycocyanin

**Fig. 3:**
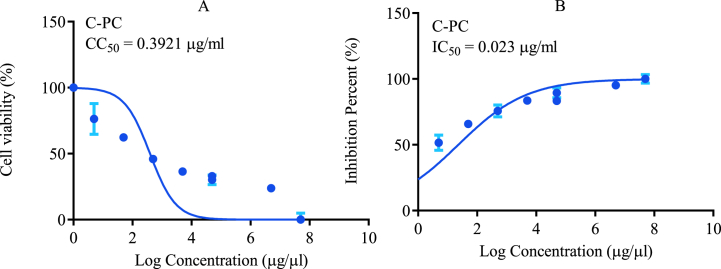
(A) Cytotoxicity (CC_50_) and (B) the 50 % inhibitory concentration (IC_50_) of C-PC against Vero-E6 cell line and human coronavirus 229E. *Log concentrations were plotted against cell viability normalized response.

Emerging viruses, such as those in the *Coronaviridae* family that cause severe acute respiratory syndrome, are becoming a growing concern for global health. It has been difficult to find effective medications against SARS-CoV-2 after more than two years of the COVID-19 pandemic. Natural, conveniently available, and cost-effective antiviral drugs that can be mass-produced in a short time are desperately needed. In this study, human coronavirus 229E was used as a model for the *Coronaviridae* family, which includes COVID-19, to assess the antiviral activity and mode of action of C-phycocyanin. Initially, the C-phycocyanin was serially diluted and introduced to cell culture media to see how it affected the growth and viability of Vero-E6 cells. The cell viability of Vero–E6 cells was evaluated using crystal violet after 3 days of incubation. The maximum non-cytotoxic concentration of C-phycocyanin was calculated to be 14.389 μg/ml. The 50 % cytotoxic and growth inhibitory doses were calculated using the mean dose-response curves of three different experiments (Fig. 3). C-phycocyanin's antiviral efficacy against HCoV-229E was tested *in vitro*. The assay used untreated virus-infected cells as a control. The 50 % inhibitory concentration (IC_50_) for HCoV-229E was found to be 0.515 μg/ml. The results are shown in Table 4 as a selective index (SI) of 27.9, which is the average of three different tests. SI for the extract against HCoV-229E was calculated using the CC_50_/IC_50_ ratio. Infectivity was lowered by more than 75.2 % at the highest non-cytotoxic C-phycocyanin concentrations. Human coronavirus replication, on the other hand, is characterized by a complex number of stages during which antiviral medications may be utilized. C-phycocyanin's mode of action wes investigated to fully understand its inhibitory effects on the HCoV-229E virus. C-phycocyanin suppressed CPE of HCoV-229E virus; when host cells were pretreated with C-phycocyanin for 1 h before infection, the C-phycocyanin showed a significant decrease in viral infection with a selective index of up to 36.76 (Table 5).

**Table 4:** Anti-Covid 19 activity of C-PC.

Sample	CC_50_	IC_50_	aSI
C-phycocyanin	14.389	0.515	27.9

a SI: Selective Index.

**Table 5:** Inhibition percentages at various phases of viral infection.

Concentration μg/ml	Inhibition %		
	Replication	Adsorption	Virucidal
5	36.1	56	43.3
0.5	30.2	47.1	28.2
0.05	22.3	45.7	9.7
0.005	15.7	36.2	2.8
0.0005	7.6	19.2	1.6

Infectivity was lowered by 56 % at peak non-cytotoxic dosages of C-phycocyanin. C-phycocyanin, on the other hand, reduced viral infection with a selectivity index of up to 4.4 when administered to host cells for 1 h after infection. Throughout the replication phase, infectivity was lowered by more than 36.1 % at maximum non-cytotoxic doses of the tested chemical. These findings not only support the preliminary screening results but also highlight the C-phycocyanin's adsorption and reproduction processes, despite the absence of antiviral efficacy throughout the virucidal mechanism (Table 6). These findings suggest that the C-phycocyanin molecule is either indirectly or directly blocking the HCoV-229E receptor position on the host cell or interfering with virion envelope structures. The findings on C-phycocyanin's antiviral activity against human coronavirus 229E are consistent with previously published data on its antiviral activity, which revealed that the antiviral activity of *S. platensis* extracts against the simplex virus (HSV-1) was significant [[60], [61], [62]]. Indeed, **Hayashi et al.** [60] discovered that a water-soluble extract of *S. platensis* decreased HSV-1 replication but had no virucidal effect or interfered with adsorption to host cells. These findings correlate with some of the findings in the present study in terms of viral suppression during replication but not during virucidal, although they disagree in terms of virus inhibition during adsorption. This compatibility and incompatibility may be due to a disparity in the structure of both viruses or *S. platensis* extracts since the present study was also conducted on human coronavirus 229E, which has an RNA genome, whereas **Hayashi et al.** [60] studied herpes virus, which has a DNA genome.

**Table 6:** Anti-proliferative IC_50_ of C-PC against HepG-2, MCF-7, and HCT-116 cancer cell lines.

IC_50_	C-PC	Doxorubicin
HepG-2	4.5^ab^ ± 0.26	5.7^a^ ±0.21
MCF-7	5.3^a^ ±0.52	5.2^b^ ± 0.43
HCT-116	4.9^a^±0.19	5.3^ab^ ± 0.16

### 3.7 Antitumor activity of C-phycocyanin

**Fig. 4:**
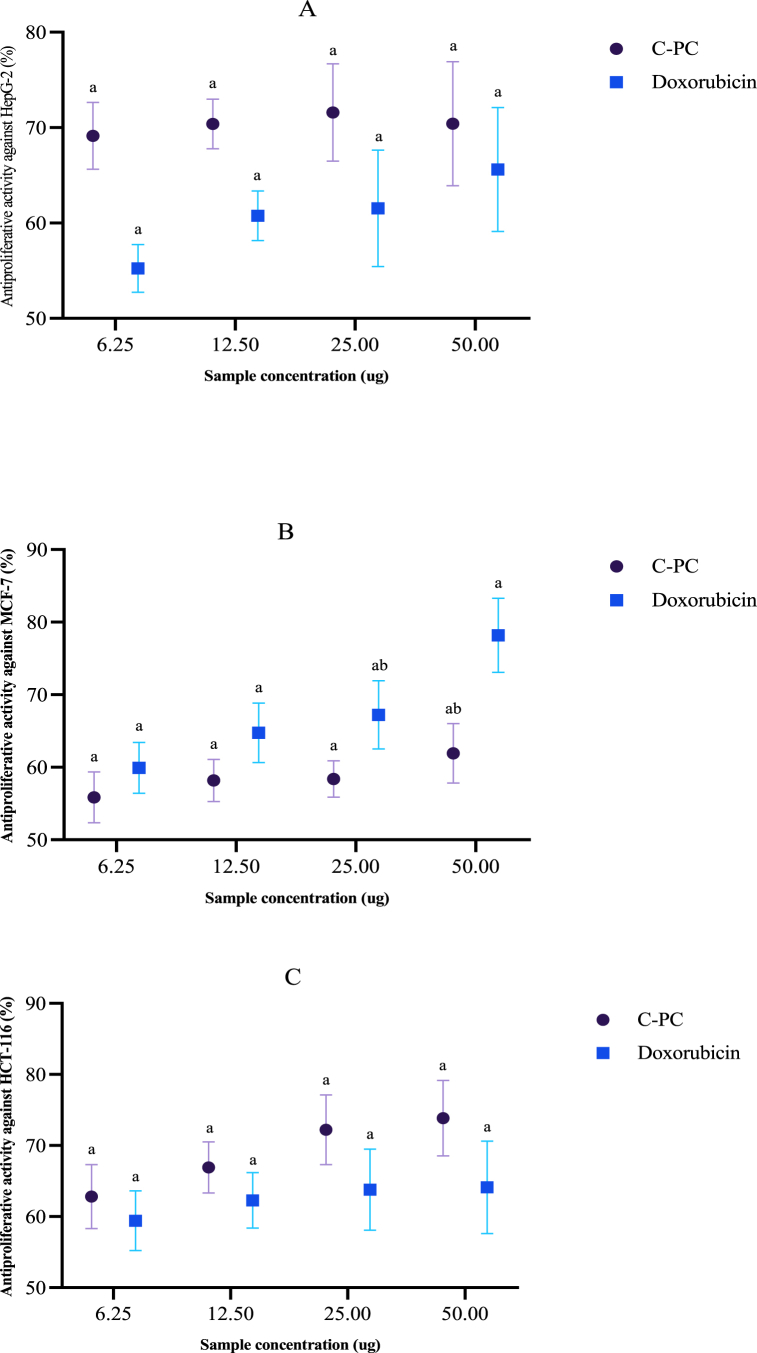
Dose dependent anti-proliferative activity of C-PC against (A) HepG-2, (B) MCF-7, and (C) HCT-116 cancer cell-lines. *Data are expressed as Mean ± SD. Similar letters are not significant while different letters are significant at *P ≤ 0.05*.

C-phycocyanin was examined *in vitro* for anti-proliferative efficacy against human cancer cell lines, HepG-2 (liver cancer), MCF-7 (breast cancer), and HCT-116 (colon cancer) using the MTT assay versus one human healthy cell line (BJ-1). The tested substance was compared to Doxorubicin's activity against the three cancer cell lines. In a dose-dependent way, C-phycocyanin inhibited all the tested cancer cells; the results showed that C-phycocyanin had potent anti-proliferative activity against liver and colon cancer, exceeding Doxorubicin and good anti-breast cancer comparable to Doxorubicin. Concerning HepG-2, human liver cancer cells, C-phycocyanin exerted notable antitumor activity up to 70 % even with the small concentration, 6.25 μg, exceeded that of Doxorubicin that reached about 55 % using the lowest concentration, 6.25 μg; and about 65 % using the highest concentration, 50 μg (Fig. 4). In the case of HCT-116, human colon carcinoma, C-phycocyanin also showed prominent anticancer activity up to 74 % using the highest concentration, 50 μg, and about 63 % using the smallest concentration, 6.25 μg, exceeded that of Doxorubicin that reached about 59 % using the lowest concentration, 6.25 μg; and about 64 % using the highest concentration, 50 μg (Fig. 4).

On the other hand, C-phycocyanin has less but still good cytotoxic activity against MCF-7, human breast cancer cells, relative to Doxorubicin. The activity reached about 62 % using the highest concentration, 50 μg, and about 56 % using the smallest concentration, 6.25 μg, compared to Doxorubicin that reached about 60 % using the lowest concentration, 6.25 μg; and about 78 % using the highest concentration, 50 μg (Fig. 4).

C-phycocyanin was also tested against non-tumor fibroblast-derived cell line (BJ) and demonstrated very low cytotoxicity. C-phycocyanin showed lower IC_50_ values than that of Doxorubicin in the case of HepG-2 and HCT-116 cell lines and moderate IC_50_ with MCF-7 cancer cell lines (Table 6).

The potent antitumor activity of C-phycocyanin reported in this study against HCT-116 and HepG-2 human cancer cells using the MTT assay with almost no cytotoxic activity towards the human healthy cell line (BJ-1) is of great interest. Using low concentrations of C-phycocyanin could exert potent anti-proliferative activity against some tested cancer cell lines, which could exceed Doxorubicin in some cases. Some chemotherapeutic medicines cause apoptosis in target cells by generating free radicals; antioxidants, on the other hand, can scavenge these radical molecules and limit the side effects of therapeutic agents [63,64].

Previous investigations reported the anti-proliferation and pro-apoptotic effects of C-phycocyanin on different cancer cell lines *in vitro* with no side effects on normal tissue cells [65]. More and more evidence has proved that C-phycocyanin has an compelling anticancer effect on various cancer cell types such as breast cancer [10], liver cancer [66], and colon cancer [67].

On the whole, 4 it was found that C-phycocyanin exhibits significant bio-therapeutic activity as an antioxidant, anti-inflammatory antitumor and *anti*-covid 19 activities that could be utilized for therapeutic purposes.

### 3.8 Production of a functional probiotic-fermented dairy product supplemented with free and nanophytosomes of C-phycocyanin

#### 3.8.1 Characterization of C-PC nanophytosomes

The impact of C-PC addition on the size and polydispersity of nanophytosomes using three different ratios from C-PC: lecithin (1:1, 1:2, and 1:3), respectively, was investigated. The C-phycocyanin nanophytosome complexes were improved by ionic interaction between the positive charge of choline groups in lecithin and the negative charge of O– and N+ groups from C-PC.

Table 7 shows the average particle size, polydispersity index, and zeta potential (surface charge) for C-phycocyanin: lecithin ratios of 1:1, 1:2, and 1:3 using dynamic laser scattering (DLS). Using a homogenizer and a probe sonicator resulted in smaller particles with transparent suspension; this condition prepared below 100 nm for all C-phycocyanin ratios (p ≤ 0.05). The size of lipid vesicles was observed to be affected by the manner of production [43].

**Table 7:** Characteristics of C-PC-loaded nanophytosomes.

C-PC: Phytosome (Molar Ratio)	Average particle size ± SD (nm)	Polydispersity Index (PDI)	Zeta (mV)	EE %	LC %
1 : 1	47.29^ab^ ± 11	0.379	−32.5 ^b^ ± 5	88.66 ^b^ ± 1.35	44.33^a^ ± 0.88
1 : 2	44.50^b^ ± 12	0.474	−32.4^b^ ± 5	94.65^ab^ ± 1.75	31.55^b^ ± 1.35
1 : 3	57.14^a^ ± 14	0.682	−27.9^a^ ± 5	97.85^a^ ± 2.15	24.46^c^ ± 0.87

C-PC: crud phycocyanin; EE: encapsulation efficiency; LC: loading efficiency; Small letters differ significantly (p ≤ 0.05) in treatments.

The particle size of C-phycocyanin nanophytosmoes 1:1 M ratio is 42.29 nm, which increased with increasing lecithin ratio of 1:2 and 1:3 to 44.50 and 57.14 nm, respectively. Also, the size distribution (PDI) for 1:1 formulation was 0.379. According to **Surini et al.** [68], high lipid composition in the formulation (1:2, and 1:3) enhanced the potential for agglomeration (PDI = 0.474, and 0.682, for 1:2, 1: 3 formulations at 25 °C).

The surface charge (ζ) of all treatments was negatively charged. The (ζ) of all formulations was between −32.50 and −27.9 mV. In general, zeta potential (ζ) values of ±30 mV were observed to prevent nanovesicles from aggregation and reduce electrostatic repulsion between nanophytosomes, which might contribute to the creation of large aggregates of nanophytosome vesicles [69]. According to the literature, negative zeta potential has been linked to the improved biocompatibility than positive zeta potential [70].

**Fig. 5:**
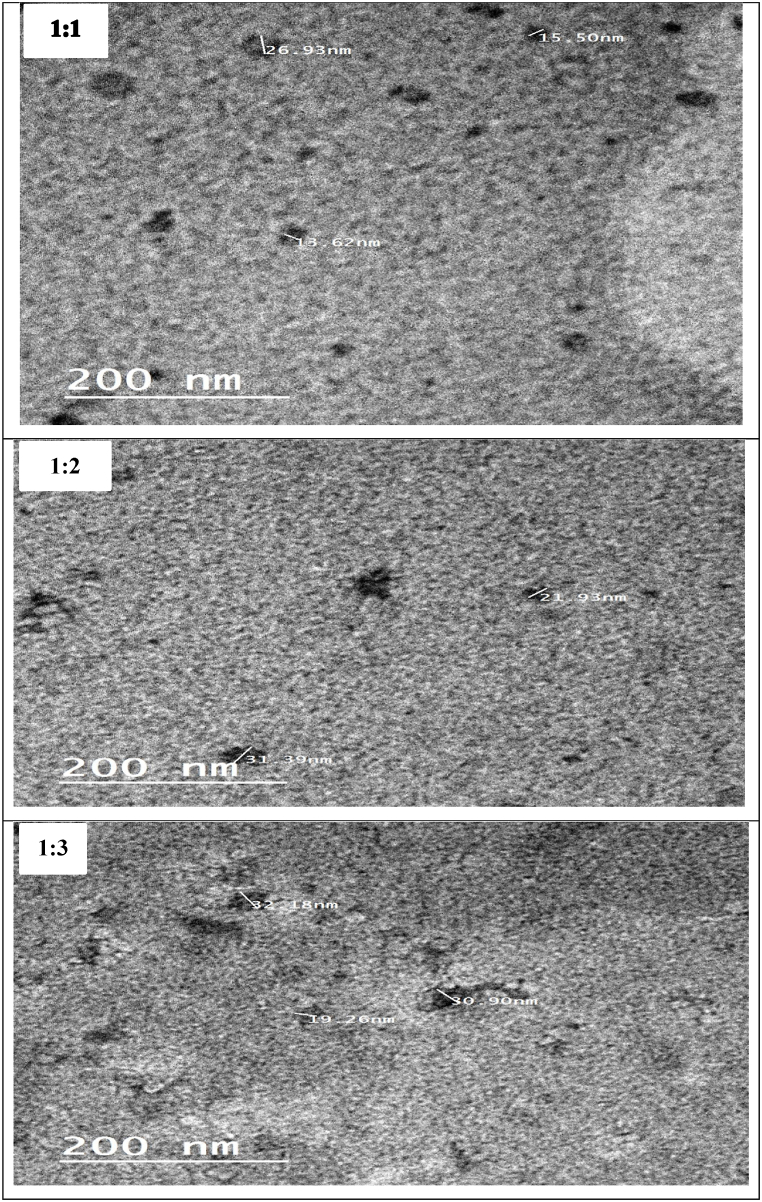
**Transmission electron microscopy of C-PC loaded nanophytosomes** C-PC: Phytosome (Molar Ratio) 1: 1, 1: 2, and 1:3.

Fig. 5 shows the TEM image of C-PC loaded nanophytosomes 1:2 formulation. The surface morphology of nanophytosomes containing C-PC confirmed the formation of spherical particles and homogeneous dispersion. The TEM image showed that the C-PC nanophytosomes were slightly homogeneous shaped and had approximately 30 nm. These results are less than DLS because of the vacuum processing steps for samples in the TEM micrograph.

The EE and LC indices are critical in evaluating the efficiency of nanocarriers to deliver bioactive components in nano-based delivery systems [71]. The experimental findings for EE and LC of C-PC in nanophytosomes were created using three formulations, revealing nanophytosomes' unique and remarkable potential as a C-PC carrier (Table 8). The maximum EE was measured for 1:1 formula (88.66 % ± 1.35), followed by 1:2 formulae (94.65 % ± 1.75) and the minimum with 1: 3 formulae (97.85 % ± 2.15). The LC for all formulations was between 44.33 and 24.46 %. The discovered systems' high LC values might be related to the usage of lecithin's inner and outer polar groups for C-PC loading, as well as lipophilic bilayer space. As previously mentioned, C-PC's interaction with the polar head of lecithin was confirmed.

**Table 8:** Chemical composition of the stirred yogurt supplemented with free and nanophytosome of C-phycocyanin.

Samples	Total solids %	Protein %	Fat %	Ash %
C	12.80^c^ ± 0.22	3.35^b^ ± 0.04	3.70^a^ ± 0.03	0.71^a^ ± 0.01
T1	12.83^c^ ± 0.21	3.57 ^ab^ ± 0.09	3.70^a^ ± 0.01	0.71^a^ ± 0.02
T2	12.86^c^ ± 0.18	3.88^a^ ± 0.07	3.65^a^ ± 0.04	0.72^a^ ± 0.01
T3	13.98^b^ ± 0.25	3.59^ab^ ± 0.03	3.65^a^ ± 0.02	0.75^a^ ±0.02
T4	15.13^a^ ± 0.17	3.97^a^ ±0.05	3.60^a^ ±0.09	0.77^a^ ±0.03

NB: All yogurt variants were fermented with *L. rhamnosus* KU985435, C: control without C-PC, T1: treatment with 150 mg free C-PC, T2: treatment with 300 mg free C-PC, T3: treatment with 150 mg C-PC nanophytosomes (at molar ratio 1:2), T4: treatment with 300 mg C-PC nanophytosomes (at molar ratio 1:2). Small letters differ significantly (p ≤ 0.05) in treatments.

#### 3.8.2 Physicochemical properties of the functional stirred yoghurt

Table 8 shows that increasing the content of free C-phycocyanin, T1 and T2, marginally enhanced mean total solids compared to control samples. In addition, total solids supplemented with nanophytosome C-phycocyanin reached a high of 13.98 and 15.13 percent at T3 and T4, respectively. This might occur because lecithin is used as an emulsifier in phytosome manufacture, which can raise total solids [72]. At fresh, the quantity of protein and ash rose dramatically when the concentration of free C-phycocyanin or C-phycocyanin-loaded nanophytosome increased.

**Fig. 6:**
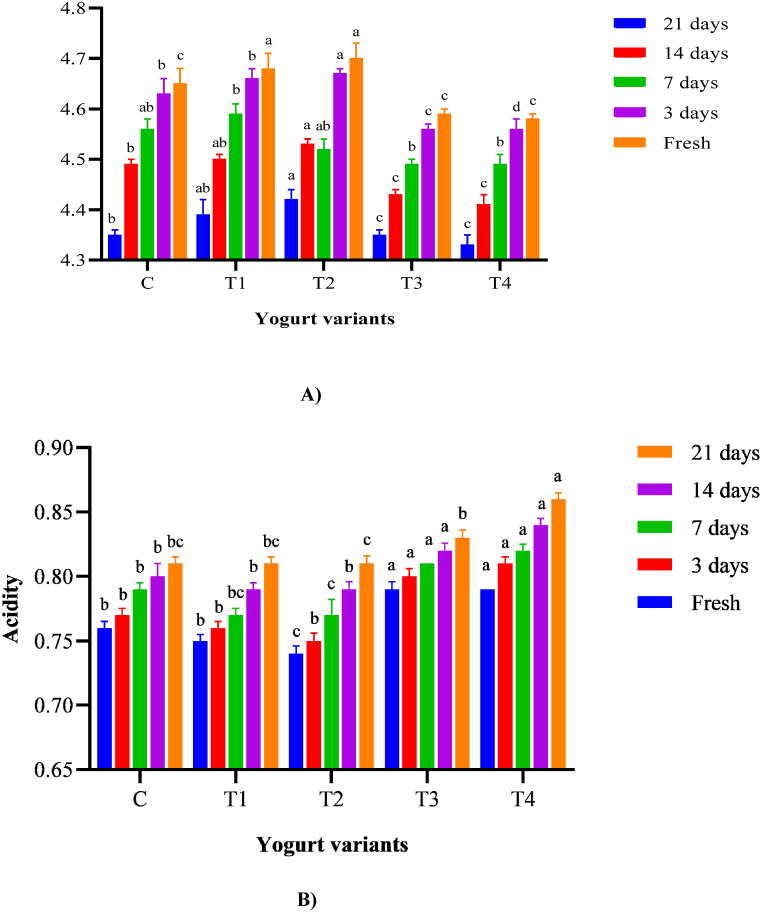
A) Changes in pH of yogurt during 21 days of storage at 5 °C Fig. 6 B). Changes in yogurt acidity during 21 days of storage at 5°C N·B: All yogurt variants were fermented with *L. rhamnosus* KU985435, C: control without C-PC, T1: treatment with 150 mg free C-PC, T2: treatment with 300 mg free C-PC, T3: treatment with 150 mg C-PC nanophytosomes (at molar ratio 1:2), T4: treatment with 300 mg C-PC nanophytosomes (at molar ratio 1:2). Data are expressed as Mean ± SD. Similar letters are not significant while different letters are significant at P ≤ 0.05.

Fig. 6 A, B show the pH and acidity variations in the yoghurt samples during 21 days of storage. The pH values of free C-phycocyanin-yoghurts were higher than the control. The elevated pH level of the C-phycocyanin-enriched yoghurt might be attributable to the C-phycocyanin pH (6.55), which demonstrates that C-phycocyanin has a significant impact. Due to the limited growth and activity of *Lactobacillus* over the 21-day storage period, neither the pH of the control yoghurt nor the pH of the C-phycocyanin-enriched yoghurt decreased considerably. As a result, C-phycocyanin did not influence starting culture development. Only little variations in pH occurred over the 21-day storage period, with minor drops until day 14 and minor rises on day 21. The increasing pH on day 21 was most likely due to the formation of metabolites such as amino acids, bacteriocins, and vitamins as the shelf life ended. During 28 days of storage, **Izadi et al.** [73] found a fall-rise pattern in yoghurt pH (P > 0.05). The metabolic activity of yoghurt starter cultures, which produces lactic acid as a consequence of lactose, causes the pH of yoghurt to drop during storage.

However, after 21 days of cold storage, the pH of stirred yoghurt enriched with C-phycocyanin nano-phytosome dropped in descending order (T4>T3) compared to other samples, likely due to the formation of lactic acid by the starting culture and probiotic bacteria. After 21 days of storage, the functional yoghurt with loaded-C-phycocyanin phytosome had a lower pH and greater acidity than the control yoghurt and that enriched with free C-phycocyanin; the findings were similar to those of **Zhong et al.** [74]. The increased starter and probiotic bacteria activity are reflected in the pH and acidity values [75].

#### 3.8.3 Viscosity

**Fig. 7:**
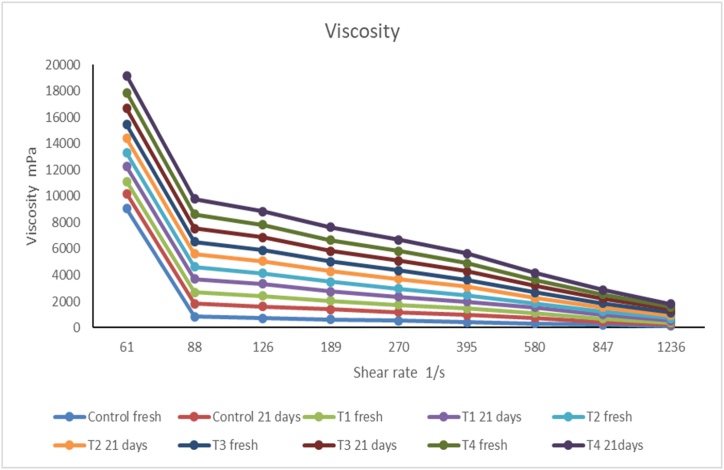
Viscosity of yogurt during 21 days of storage at 5°C N·B: All yogurt variants were fermented with *L. rhamnosus* KU985435, C: control without C-PC, T1: treatment with 150 mg free C-PC, T2: treatment with 300 mg free C-PC, T3: treatment with 150 mg C-PC nanophytosomes (at molar ratio 1:2), T4: treatment with 300 mg C-PC nanophytosomes (at molar ratio 1:2).

As reported in Fig. 7, the viscosity of stirred yoghurt fortified with free C-phycocyanin was slightly higher than that of plain set yoghurt because C-phycocyanin probably brought about changes in the microstructure of yoghurt gel. The resulting higher shear stress increased their viscosity compared with the control. On the other hand, the viscosity increased with increasing C-phycocyanin concentration due to the increased total solid content of yoghurts; clearly, solid content is one-factor affecting viscosity. **Mohameed et al.** [76] found that increasing solid content would increase product viscosity.

While the apparent viscosity of yoghurt increased dramatically with the increased addition of C-phycocyanin nanophytosome compared to the control, this is due to the absorption of water by lecithin used in the preparation of phytosome, which would increase the viscosity. **Daba et al.** [77] found an increased viscosity of yoghurt fortified with nano phytosome of *Hydnora abyssinica* extraction compared with plain yoghurt. Also, **Ghorbanzade et al.** [72] found that the increase of the total solids in yoghurt with added nano-capsules may be responsible for the increased viscosity. The findings are consistent with those of **Hamed et al.** [78], who found that the viscosity of stirred yoghurt held at 5 °C increased from day 1 to day 7. They ascribed the rise to post-acidification and mild but sustained metabolic activity at 5 °C till day 7.

#### 3.8.4 Sensory evaluation of the functional stirred yoghurt

**Fig. 8:**
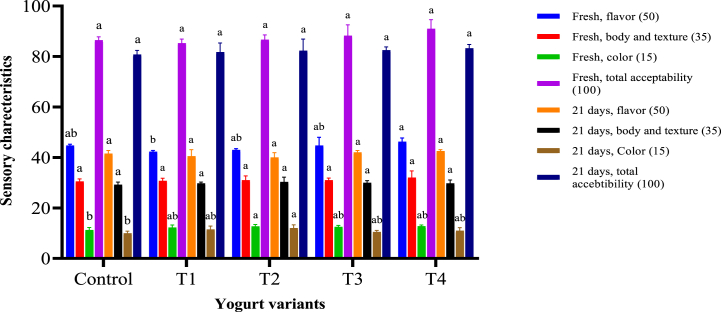
Sensory evaluation of yogurt during 21 days of storage at 5°C N·B: All yogurt variants were fermented with *L. rhamnosus* KU985435, C: control without C-PC, T1: treatment with 150 mg free C-PC, T2: treatment with 300 mg free C-PC, T3: treatment with 150 mg C-PC nanophytosomes (at molar ratio 1:2), T4: treatment with 300 mg C-PC nanophytosomes (at molar ratio 1:2). Data are expressed as Mean ± SD. Similar letters are not significant while different letters are significant at P ≤ 0.05.

Fig. 8 illustrates the findings of the sensory evaluation. On the first day, all yoghurt samples were reviewed and found to be similar in flavor, body and texture, color, and overall acceptance. The panelists assigned the C-phycocyanin nano-phytosome yoghurts the highest marks for body and texture. However, there were no significant changes in body and texture between the control stirred yoghurt and the yoghurt fortified with free C-phycocyanin. The panelists gave higher color scores to yoghurt fortified with free C-phycocyanin (150 mg) than 300 mg. However, the flavour of the yoghurts with 150 and 300 mg of C-phycocyanin was not significantly different from the control. Based on sensory assessments, the panelists deemed the flavour of yoghurts with C-phycocyanin concentrations of up to 300 mg satisfactory, especially when encapsulated with phytosomes.

Furthermore, the nanophytosome C-phycocyanin yoghurt 300 mg had higher overall acceptance and showed no significant difference from the control yoghurt. For the last 30 years, *Spirulina* microalgae have been used as a supplement in various health foods and have been commercially produced for this reason. *Spirulina* is regarded as a high-quality food and nutraceutical supplement because of its high protein, fat, vitamins, minerals, chlorophyll, beta-carotene, and polysaccharides. *Spirulina*'s usage has been correlated with customer understanding of the value of natural color agents because of its nutritional, pharmacological, and health-related advantages due to its distinctive natural color, C–C-phycocyanin. As a result, natural pigments and *Spirulina* as a source of color are becoming increasingly popular, notably in the food and cosmetics industries [78].

#### 3.8.5 Determination of shelf-life stability of stirred yoghurt

Control yoghurt samples turn spoiled after 5 days of incubation, while remaining samples (inoculated with *Lactobacillus rhamnosus* KU985435 and supplemented with C-PC free and nano-encapsulated phytosomes) had a longer storage time, they remained unspoiled for more than one month indicating that *Lactobacillus rhamnosus* KU985435 and C-PC prolonged the storage time (validity) of the yoghurt. Moreover, the present study showed that all the tested variants of fermented milk were characterized by good content of live probiotic bacteria (at least 10^10^ CFU/ml) also during refrigerated storage, with an increase in their number observed, assuring the level of the therapeutic minimum (Table 9). The increased viability of probiotic bacteria during storage may be due to the presence of the prebiotic promising C-PC and could also be due to that pH of the product did not decrease greatly during storage. **Sady et al.** [79] explained the decrease in the number of probiotics by the increase in the acidity of the samples during storage.

**Table 9:** Shelf life stability (probiotic count, total phenolic and total antioxidant) of the stirred yogurt.

Yogurt samples	CFU*10^10^/ml			Total phenolic (GAE mg/g)			Total antioxidants (GAE mg/g)						C-PC Remain (%)
							DPPH			ABTS			
	24 h	7 days	14 day	24 h	7 days	14 day	24 h	7 days	14 day	24 h	7 days	14 day	After 14 days
C	3.47^Cab^	5.83^Ba^	7.32^Ab^	226.00^Ce^ ± 18.38	307.50^Ae^ ± 6.36	271.50^Be^ ± 9.19	59.51^ABe^±2.31	63.99^Ad^ ±2.74	58.99^Bd^ ±3.17	42.40^Bd^ ±1.51	75.13^Ae^ ± 1.57	71.68^ABe^ ±0.73	–
T1	3.86^Ca^	5.94^Ba^	8.83^Aa^	685.00^Bd^ ±19.80	775.50^Ad^±10.61	789.50^Ad^ ±14.85	64.69^Ad^±4.32	62.20^Ad^ ±5.56	58.00^BAd^ ±4.72	62.65^Cc^ ±7.33	129.41^Ad^±3.64	123.09^Bd^±1.79	35.77^b^ ± 3.25
T2	3.35^Cb^	5.71^Bab^	8.24^Aab^	975.50^ABc^ ±7.78	984.50^Ac^±7.78	907.50^Bc^±10.61	64.04^Bc^±2.35	69.01^Ac^ ±4.56	61.17^BCc^ ±5.25	75.37^Cb^ ±1.33	161.12^Ac^±2.63	148.67^Bc^±15.36	39.08^b^ ± 2.85
T3	1.77^Cc^	2.68^Bb^	4.21^Ac^	1132.50^Cb^±17.68	1280.00^Bb^ ±12.73	1306.50^Ab^ ±2.12	94.36^Bb^±3.89	108.40^Ab^ ±4.02	109.45^Ab^ ±3.23	85.73^Cab^ ±4.00	189.66^Bb^±2.21	198.48^Ab^±2.38	95.25^a^ ± 3.75
T4	1.91^Cc^	2.74^Bb^	4.46^Ac^	1283.50^Ca^±13.44	1444.50^Ba^ ±9.19	1562.50^Aa^ ± 9.19	104.98^Ba^ ±3.96	127.21^Aa^ ±5.09	128.88^Aa^ ±3.82	89.97^Ba^ ±4.66	203.74^Aa^ ±4.12	201.38^Aa^±1.24	93.45^a^ ± 2.58

NB: All yogurt variants were fermented with *L. rhamnosus* KU985435, C: control without C-PC, T1: treatment with 150 mg free C-PC, T2: treatment with 300 mg free C-PC, T3: treatment with 150 mg C-PC nanophytosomes (at molar ratio 1:2), T4: treatment with 300 mg C-PC nanophytosomes (at molar ratio 1:2). Values represent mean *±* SD for three independent experiments. Small letters differ significantly (*p* ≤ 0.05) in treatments and capital letters between storage periods.

The stirred yoghurt's total phenolic and antioxidant capacity, which refer to phycocyanin stability, was determined periodically over 14 days of storage. Phytochemicals in phytosomal structures are mostly contained inside vesicles, resulting in increased bioavailability due to enhanced permeability to membrane and cell absorption. Phosphatidylcholine expands when in contact with water, forming a hydrogel layer. This layer gradually releases the core material (C-PC) to the outer surface. Gong et al. [80] found that the mechanism of phosphatidylcholine leads to the extended preservation of C-PC nanophytosomes in acidic yoghurt settings. Phycocyanin can be extracted as previously mentioned in methods 2.2.2, 2.2.6, and 2.4.2.3.2. Thus, it is possible to determine the remains of C-PC, total phenolic content, and antioxidant capacity, which refer to phycocyanin remains. Encapsulated C-PC enriched yoghurt (T3 and T4, using a concentration of 150 and 300 mg) showed the maximum total phenolic content compared to other treatments, including plain yoghurt. DPPH and ABTS radical scavenging methods demonstrated that free and encapsulated C-PC-enriched yoghurts showed a notable increase in the percentage of inhibition compared to plain yoghurt. There is a significant difference between free and encapsulated C-PC fermented samples. C-PC encapsulated yoghurt showed a high capacity to scavenge the DPPH and ABTS radicals due to the remains of C-PC in yoghurt.

Interestingly, total phenolics were not negatively affected by refrigerated storage; in contrast, phenolic contents increased by increasing storage time. Also, there is an increase in TPC and antioxidant capacity with storage period due to the protein peptides in fermented milk. Daba et al. [77] found that the enhanced ability to remove free radicals was attributed to the protein peptides obtained from fermented milk. Fermented milk has significant radical scavenging properties, indicating its potential as a natural antioxidant supplement for enhancing human health. Balakrishnan & Agrawal [81] found that milk fermented with probiotic bacteria with more excellent antioxidant activity may increase the antioxidant capacity of the resulting product.

The results showed the lowest remaining C-PC in T1 and T2, and the protein of C-PC might be highly degraded by starter strains and acidic conditions. The remains of C-PC in T3 and T4 showed high stability and low degradation, possibly due to the encapsulation by phytosomes, which gives high protection. Previous research demonstrated that the change in the blue color of PC is caused by the denaturation of the protein structure, which is triggered by either acid or alkaline conditions. This denaturation disrupts the organisation and transmission of energy among the tetrapyrrole chromophores contained in the protein [82].

A functional fermented dairy product must pass through the storage environments with minimum loss. Therefore, the abovementioned results could confirm the viability and validity of the prepared yoghurt for over 14 days, the standard period for refrigerated yoghurt storage with the stability of C-PC. Antioxidant components in food play an important role as a health-protecting feature. Scientific evidence suggests that antioxidants reduce the risk of chronic diseases, including cancer and heart disease [83].

## 4 Conclusion

As related to the topical interest of public health professionals about functional food products from renewable, sustainable sources and their biotherapeutic activity, C–C-phycocyanin (C-PC) from the blue-green edible algae *Spirulina platensis* was extracted and characterized for its biotherapeutic potential. The results proved the efficacy of C-phycocyanin as an antioxidant, anti-inflammatory, antitumor, and antiviral active protein, which could be investigated as a novel therapeutic option for *Coronaviridae* viruses such as COVID-19. Most people find algal extracts unacceptable and susceptible to denaturation and/or proteolysis, so they are encapsulated in a complex nanophytosome. Nano-encapsulation of C-PC could cover unacceptable taste and odor and protect its biological activity to produce functional yoghurt with a potent probiotic, *Lactobacillus rhamnosus* KU985435 and supplemented with free or nanophytosomes of C-phycocyanin. The produced yoghurt showed the desired physicochemical, microbiological properties, and sensory acceptance when fortified with the nanophytosome of C-PC and high antioxidant capacity to scavenge DPPH and ABTS radicals. In conclusion, the nanophytosome technique gives C-PC stability to be applied in an innovative functional food product and further utilized in natural drug development to enhance their bilogical activity.

## Funding

This research did not receive any specific grant from funding agencies in the public, commercial, or not-for-profit sectors.

## Ethical approval

The study was conducted in accordance with the ethical Procedures and policies approved by Animal Care and Use Committee of National Research Centre, Egypt, which complies with the guidelines from the Canadian Council on Animal Care. Approval Number 12050205.

## Data availability statement

All data obtained from this research work were included and available in the manuscript.

## CRediT authorship contribution statement

**Tarek Nour Soliman:** Writing – review & editing, Writing – original draft, Visualization, Supervision, Software, Project administration, Methodology, Funding acquisition, Formal analysis, Conceptualization. **Asmaa Negm El-Dein:** Writing – review & editing, Writing – original draft, Visualization, Supervision, Methodology, Formal analysis, Conceptualization. **Sahar AbdAlDiam:** Writing – original draft, Visualization, Methodology, Formal analysis. **Abdou Allayeh:** Writing – original draft, Validation, Methodology, Data curation. **Hanem Awad:** Writing – original draft, Visualization, Methodology, Investigation, Conceptualization. **Nasser S. Flefil:** Writing – review & editing, Writing – original draft, Supervision, Methodology, Investigation, Formal analysis, Conceptualization.

## Declaration of competing interest

Formulation of C-PC nanophytosomes was Patent registered under the No: EG/P/2022/1221.
